# Soil-transmitted helminths and schistosomiasis among pre-school age children in a rural setting of Busia County, Western Kenya: a cross-sectional study of prevalence, and associated exposures

**DOI:** 10.1186/s12889-020-08485-z

**Published:** 2020-03-18

**Authors:** Janet Masaku, Doris W. Njomo, Ann Njoka, Collins Okoyo, Faith M. Mutungi, Sammy M. Njenga

**Affiliations:** Eastern & Southern Africa Centre of International Parasite Control (ESACIPAC), Kenya Medical Research Institute (KEMRI), Mbangathi road, P.O.Box 54840-00200, Nairobi, Kenya

**Keywords:** Pre-school age children, Soil transmitted Helminths, Risk factors

## Abstract

**Background:**

Soil-transmitted helminths (STH) and schistosomiasis continue to cause serious health problems among affected communities. To ensure that infection transmission levels are reduced, repeated mass drug administration at regular intervals has been recommended by World Health Organization. Pre-school age children (PSAC) have been neglected both in terms of research activities and in control programmes in the past for reasons that they carry insignificant infection levels. The current study determined risk factors that contribute to differences in infection prevalence among enrolled and non-enrolled PSAC in Busia County, western Kenya.

**Methods:**

This was a comparative cross-sectional study that compared STH and *Schistosoma mansoni* infections among enrolled and non-enrolled PSAC in Busia County. Simple random sampling was used to select study participants. A total of 327 enrolled and 326 non-enrolled PSAC (*n* = 653) were recruited from five participating schools, and the neighboring villages. Statistical analysis was performed using STATA version 14 (STATA Corporation, College Station, TX, USA). Differences in proportions were assessed using the z-test statistic for testing sample proportions. Univariable and multivariable logistic regression were used to test the associations between the variables.

**Results:**

The prevalence of STH and *Schistosoma mansoni* infection was 17.0% (95%CI: 13.1–22.1), and 11.8% (95%CI:11.0–12.9) respectively. Specific STH species prevalence were 12.9% (95%CI:7.0–23.5) for *Trichuris trichiura*, 8.3% (95%CI:8.2–8.3) for *Ascaris lumbricoides,* and 1.2% (95%CI:1.2–1.2) for hookworms. Prevalence of *T. trichiura* was higher among enrolled PSAC 16.9% (95%CI: 6.8–41.9); *p* = 0.003, compared to the non-enrolled 8.9% (95%CI:4.3–18.2). From univariable analysis, lack of improved water source for drinking OR 2.01, (95%CI:1.29–3.13); *p* = 0.002, and not wearing shoes OR 3.42, (95%CI:1.14–10.29); *p* = 0.028, were some of the significant factors associated with STH infection. While for multivariable analysis, bathing/swimming in a river daily, aOR 3.99 (95%CI:1.98–8.06); *p* = 0.001 was a key significant factor for *S. mansoni* infections.

**Conclusion:**

There was high prevalence of STH infection among enrolled PSAC despite having participated in the national school-based deworming programme. Hence the need for continued mass drug administration to reduce the intensity of infections among these age group. In addition, further research maybe needed to identify drivers of STH infection particularly *T. trichiura* among PSAC.

## Background

Soil-transmitted helminths (STH) and schistosomiasis are among the world’s neglected tropical diseases (NTDs) [1]. In the Global Burden of Disease Study in year 2010, NTDs accounted for 26.06 million disability-adjusted life years (DALYs) [2]. STH and schistosomiasis form a vicious cycle, whereby they contribute to poverty in endemic communities [1, 3]. The morbidity due to STH infections is greatest in school-age children (SAC) who typically have the highest intensity of infections [4, 5]. In Kenya, STH mostly occur in western and coastal Kenya and selected foci in other parts of the country [6, 7]. Growth stunting, iron-deficiency anemia, rectal prolapse, and chronic dysentery are some of the features of STH and schistosomiasis infections. These parasitic diseases also adversely affect to a great extend the cognitive development, loss of appetite, reduced nutrient absorption, and iron loss in childhood [8].

During the 66th World Health Assembly (WHA) in May 2013, the meeting resolved to intensify, and integrate measures against NTDs, through resource mobilization to improve health of affected populations [9]. Pre-school age children (PSAC), defined as children aged from 3 to 5 years have shown to be particularly vulnerable to these infections [8, 10, 11]. Although, children belonging to this age group are less likely to harbor heavy infections, the infections can lead to high risk of anemia and wasting malnutrition [12]. It is estimated that 21 million PSAC are infected with hookworms, 122 million are infected with *A. lumbricoides* and 86 million are infected with *T. trichiura* [13]. Although these infections are not among the top leading causes of death, they endanger children’s health in a subtle and debilitating way.

To ensure that infection transmission levels of schistosomiasis is reduced, repeated mass drug administration (MDA) on affected populations at regular intervals is recommended by World Health Organization (WHO) [14, 15]. Furthermore, WHO recommends once per year MDA in areas of low risk of STH infection for PSAC [16]. While in areas of high-risk prevalence (> 50%) bi-annual treatment is recommended, as well as the promotion of access to safe water, sanitation, and health education through intersectoral coordinated activities [16, 17]. Albendazole, mebendazole, levamisole, and pyrantel pamoate are considered essential medicines for the treatment of hookworms, *A. lumbricioides*, and *T. trichiura* [18]. The drugs albendazole and mebendazole are particularly recommended by WHO, to control morbidity among children living in endemic areas, for regular large-scale MDAs [19]. In Kenya, schistosomiasis is predominantly caused by *Schistosoma mansoni* and *Schistosoma haematobium.* Previous studies have shown that there is a direct relationship between the prevalence of infection and distance to Lake Victoria [20]. *S. mansoni* is mostly found in western Kenya, and some selected foci in central region, while in the coastal areas, schistosomiasis is exclusively caused by *S. haematobium* [7, 21].

In Kenya, MDA is given to children by trained primary school teachers under a National School-Based Deworming Programme (NSBDP) [22]. Targeting anthelminthic to SAC capitalizes on the fact that the heaviest burdens of infection are found in this portion of the population [17]. While school-based treatment is currently a priority, non-enrolled PSAC also experience substantial morbidity, and may benefit from deworming but are currently left out unintentionally in some cases [6, 23]. This may increase their risk of developing morbidity in future, which may have negative impacts on the overall effectiveness of current control programmes [17, 24].

Currently, there is little data on the epidemiology of STH and schistosomiasis infection among PSAC, and the associated risk factors. Such data is pertinent for control of STH among PSAC who are at risk of morbidity and may add up as reservoir of the parasitic infection. Some of the risk factors for STH infection shown by previous studies are, low socioeconomic status, rural residency, and poor sanitation [4, 25]. Improved drinking water, and proper human waste disposal through use of pit latrines, has been associated with reduced STH prevalence [26]. Nevertheless, maternal STH infection has been associated with increased STH infection, indicating the need of identifying controllable risk factors during PSAC targeted interventions [27].

Increased knowledge of the health effects for these infections, can provide precise estimation of the disease burden, by understanding the risk factors, and assessment of their extent [6]. Such information may give guidance on combined, and integrated control strategies for specific epidemiological settings [21, 28, 29]. These risk factors include deworming history, individual characteristics, household characteristics, sanitation behaviors, and practices. Previous studies have shown that some of the correlates like consumption of uncooked meat, maternal education, bathing/swimming in fresh water bodies, and consumption of un boiled/un treated water are among the associated risk factors [30–32]. This study investigated several factors that may contribute to differences in infection prevalence of STH, and schistosomiasis in Busia County among PSAC.

## Methods

### Study design

This was a cross-sectional comparative study involving collection of parasitological data, and exposures of STH and *S.mansoni* infection. A structured questionnaire was used to collect data from parents/guardians of the PSAC aged 3–7 years old. The study was conducted in the months of January and February 2018 prior to the annual school-based deworming exercise in Bunyala Sub-County, Western Kenya. Bunyala Sub-County was purposively sampled owing to previous studies done in the area, which demonstrated high prevalence of STH and *S.mansoni* infection [33]. Five primary schools which had been participating in the NSBDP were purposively selected for the study [22]. In addition, surrounding villages which are within a radius of 5 Km were also selected for recruitment of non-enrolled PSAC.

### Study population

The target population was 700 PSAC (3–7 years old) from two cohorts selected from five primary schools (enrolled PSAC), and the surrounding villages (non-enrolled PSAC). The study was conducted in a rural setting, whereby children above 5 years were in pre-school or yet to enroll. Enrolled PSAC were children attending preschool classes in the selected primary schools. While, non-enrolled PSAC were children who qualified for enrolment in pre-school based on age (3 years). But delay due to various reasons like long distance to school, and lack of school uniform or fees due to poverty.

### Study area

Busia County, in Western Kenya borders Kakamega County to the east, Bungoma County to the north, Lake Victoria to the south and Uganda to the west. The climate is composed of an average temperature of 22 °C, and the rainfall amount ranges between 750 mm and 1800 mm per annum. The County has a population of 743,946 densities with 439 people per Km^2^. The County economy is heavily reliant on farming, sugarcane being the principal cash crop [34]. In Bunyala Sub County, STH and *S. mansoni* are co-endemic with the latter being prevalent around the lake, and irrigation area where rice farming is done [22].

### Sample size determination, and sampling procedure

The sample size calculation was done to estimate the number of study participants to be selected in each school**,** and village. The unit of analysis was prevalence of infection among enrolled and non-enrolled PSAC, within the sampled schools**,** and villages respectively. Simple random sampling was used to recruit 60 (plus 10 reserves) PSAC from each participating primary school to make a total of 350. This was done using computer generated numbers from excel spread sheet. Since there were 5 catchment schools, 70 non-enrolled PSAC (including 10 reserves) were recruited from each selected village based on practical feasibility. Systematic sampling was conducted where every forth household with non-enrolled child was included. Each study village was mapped, divided into sectors based on topography. To achieve this, the list of the total number of households in each village was obtained from the local village heads**,** and area chief. Houses with non-enrolled PSAC were numbered**,** and each child registered according to household. PSAC who were sick, failed to provide stool sample, had received deworming during the last 6 months or whose parents/guardians were absent at the time of the survey were excluded.

### Sample collection

In the school setting, parents/guardians of the PSAC who had been selected as study participants, where requested to accompany their children on the specified day of sample collection. The research team presented information about the study during group meetings, and thereafter written consents were sought from parents/guardians of the selected children. The selected PSAC were issued with a stool container and accompanied by their parents/guardians to the latrines for stool collection with the help of trained research assistants**,** and the early childhood development (ECD) teacher. Questionnaire guide interviews were conducted with parents/guardians of the selected study children. The collected stool samples were transported to Port Victoria/ Bunyala sub-county hospital for analysis.

### Stool sample processing using Kato-katz technique

Fresh morning stool samples were collected from participating PSAC. Screening for STH and schistosome ova was based on duplicate thick smears consisting of 41.7 mg stool prepared using the Kato-Katz technique [35]. The slides were examined under a microscope within 30 min and the counts expressed as eggs per gram (epg) by multiplying with a standard conversion factor of 24. The parasitological procedure was carried out by trained medical laboratory technologists from the Ministry of Health (MOH). Any resulting discrepancies in the slide readings were resolved by a senior technologist. For quality assurance (QA) purposes, 10% of the slides were re-read by a senior technologist.

### Questionnaire survey

A structured pretested questionnaire developed in English and translated into local dialect was administered to all the parents/guardians of PSAC by trained research assistants. The questionnaire was structured to collect demographic information (age, gender**,** and name of parent/guardian) and socio-economic status of parents/guardians. The socio-economic indicators included level of education, occupation, and property owned. Information on water, sanitation**,** and hygiene (WASH) was also captured in a separate section. Other environmental indicators that were assessed included presence of a pit latrine at home, presence of hand washing facility, source of water for drinking in their homestead**,** and preferred place of defecation. In addition, bathing or swimming in fresh water bodies**,** wearing shoes, eating soil/clay, receiving deworming drugs, and any noticeable abdominal pain on their PSAC.

### Statistical analysis

Data was collected and entered into smartphones using open data kit (ODK), downloaded to Microsoft Excel Spreadsheet, cleaned**,** and exported for analysis. All statistical analyses were carried out using STATA version 14.1 (STATA Corporation, College Station, TX, USA). Prevalence was defined as the percentage number of individuals positive for any of the infections over the number examined. While infection intensity was defined as the average number of egg counts expressed as arithmetic mean eggs per gram of feaces (epg). Prevalence, and average intensity of infections were calculated for STH, and *S. mansoni*. 95% confidence intervals (CIs) were obtained using binomial, and negative binomial regression models respectively considering clustering at school or village levels. We used the negative binomial model since the distribution of egg counts was over dispersed. Further, we compared the prevalence between the enrolled and non-enrolled groups of PSAC using the z-test statistic for two-sample test of proportions reporting the z-test statistic and the associated *p*-values. Analysis of factors associated with either STH or *S. mansoni* infections was initially analyzed using univariable analysis and described as odds ratio (OR) using logistic regression. For multivariable analysis, minimum adequate variables were selected using forward step-wise variable selection method, specifying an inclusion criterion of *p*-value < 0.2. Adjusted OR (aOR) were then obtained by mutually adjusting all the minimum generated variables using multivariable logistic regression model.

## Results

### Demographics

A total of 653 children participated in the study, with 327 (50.1%) being enrolled PSAC surveyed from 5 primary schools**,** and 326 (49.9%) being non-enrolled PSAC from 5 villages around the sampled schools. Mean age was 4.8 years (range: 3–7 years, SD = 1.3 years)**,** and male children accounted for 50.5% (330/653). Parents/guardians of PSAC were mostly engaged in small businesses (42.4%), and farming (38.0%) as the main occupation. Assessment of the household structures showed that most houses had iron sheets roofing (73.7%), with mud/earthen floors (62.8%)**,** and walls (68.8%). The reported deworming rate was low at 349 (53.5%) among all the children. Comparatively, non-enrolled children from the villages had the least deworming rates at (44.2%) than the enrolled PSAC who had deworming rate at (62.7%).

### Prevalence, and intensity of STH, and *S. mansoni* infections

**Fig. 1:**
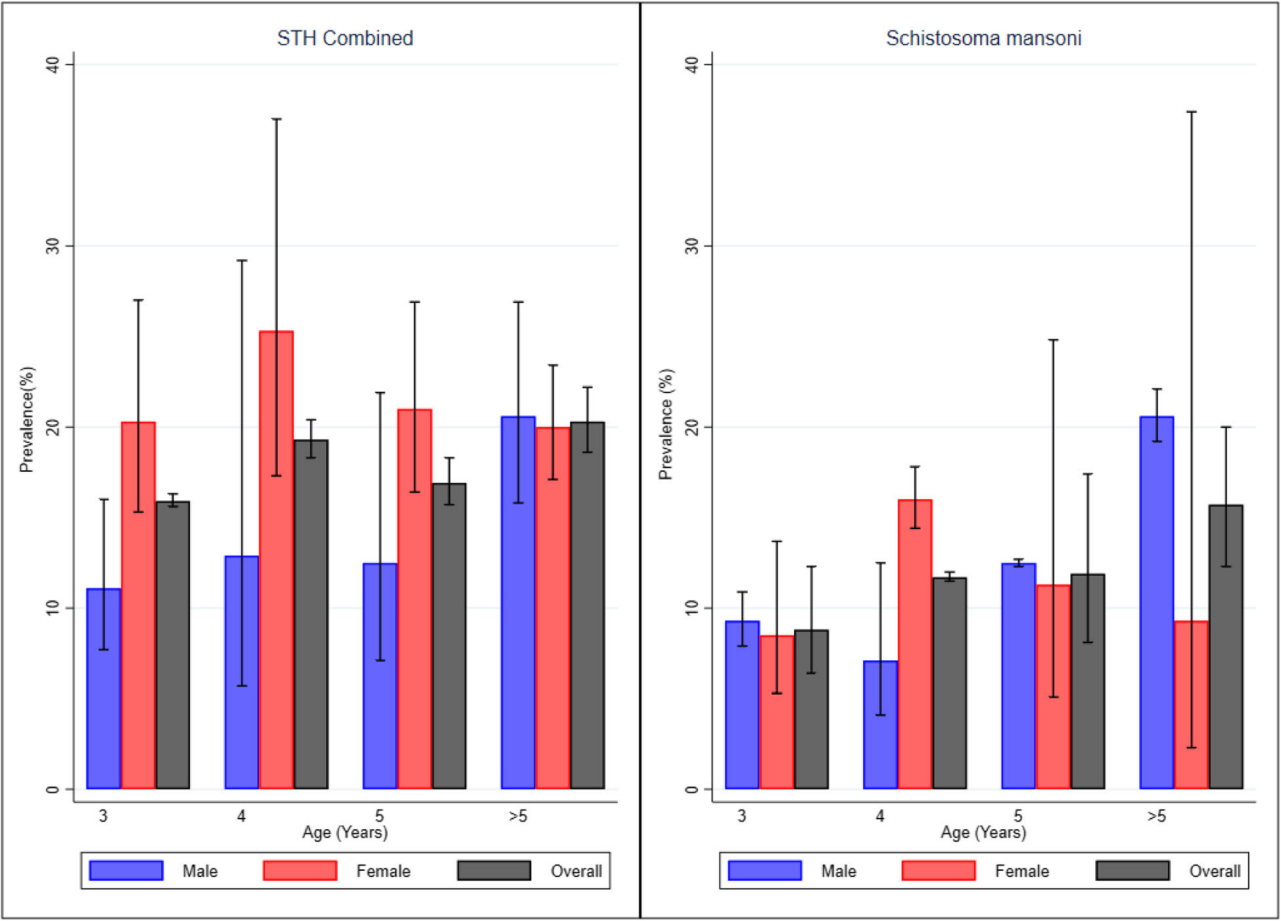
STH and *Schistosoma mansoni* prevalence (%) by age and gender among the pre-school age children in Busia County, Kenya

The overall prevalence for STH infection was 17.0% (95%CI: 13.1–22.1) with specific species prevalence of 12.9% (95%CI: 7.0–23.5) for *Trichuris trichiura*, 8.3% (95%CI: 8.2–8.3) for *Ascaris lumbricoides* and 1.2% (95%CI: 1.2–1.2) for hookworms (Table 1). The overall mean intensity for STH infection was 870 eggs per gram (epg) (95%CI: 867–872) with *A. lumbricoides* having the highest mean intensity of infection followed by *T. trichiura* and then hookworms. Female PSAC had higher STH prevalence of 19.8% (95%CI: 15.9–24.7) compared to males 14.2% (95%CI: 10.9–18.6). Prevalence of *S. mansoni* among male PSAC was 12.4% (95%CI: 9.3–16.5), and female PSAC was 11.1% (95%CI: 8.2–15.2). The overall STH prevalence increased with age. PSAC aged 3 years had the least prevalence of 15.9% (95%CI: 15.6–16.3), while those aged 7 years had the highest prevalence of 20.6% (95%CI: 19.8–21.5). Similarly, the prevalence of *S. mansoni* increased with age, with PSAC aged 3 years showing the least prevalence of 8.8% (95%CI: 6.4–12.3) while those aged 7 years with 20.6% (95%CI: 12.3–34.6) prevalence (Fig. 1). Even though, infection intensity for both STH and *S. mansoni* were highest among 7 years PSAC, it did not follow a clear pattern of progression with increasing age.

**Table 1:** STH and *S. mansoni* overall prevalence (%), prevalence comparison between non-enrolled and enrolled children, and two-sample test of proportions of prevalence among pre-school aged children in Busia County, Kenya

Infections	Overall (*n* = 653)	Enrolled children (*n* = 327)	Non-enrolled children (*n* = 326)	Two-sample test of proportions (Z-test, *p*-value)
STH Combined	17.0 (13.1–22.1)	19.3 (8.6–43.5)	14.7 (9.1–23.9)	Z = 1.545; *p* = 0.122
Hookworms	1.2 (1.2–1.2)	1.2 (0.5–3.0)	1.2 (0.5–3.1)	Z = -0.004; *p* = 0.997
*A. lumbricoides*	8.3 (8.2–8.3)	8.3 (3.0–22.6)	8.3 (4.7–14.5)	Z = -0.012; *p* = 0.991
*T. trichiura*	12.9 (7.0–23.5)	16.9 (6.8–41.9)	8.9 (4.3–18.2)	Z = 3.024; *p* = 0.003
*S. mansoni*	11.8 (11.0–12.9)	12.3 (7.0–21.6)	11.3 (5.2–24.8)	Z = 0.350; *p* = 0.727

**Fig. 2:**
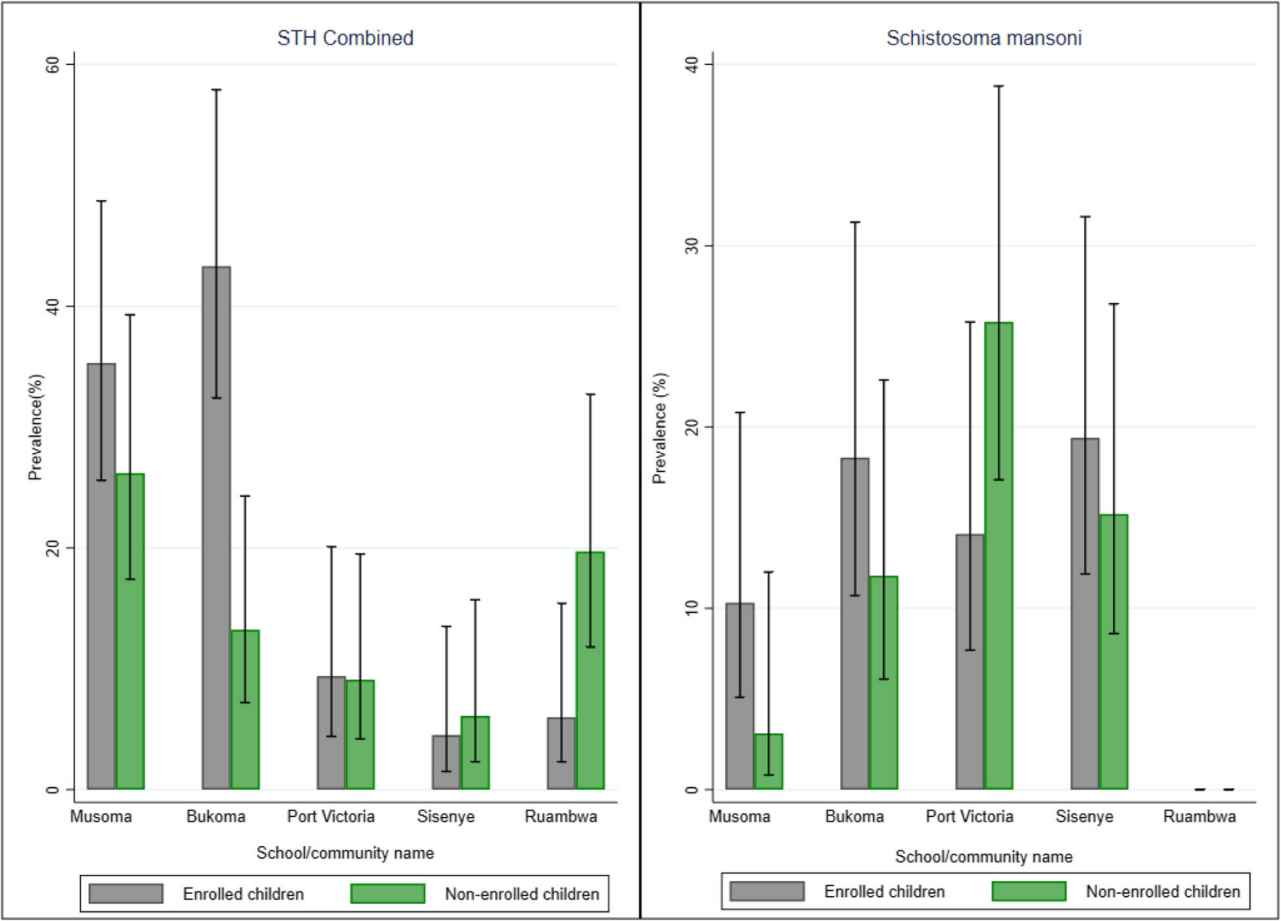
STH and *Schistosoma mansoni* prevalence (%) among enrolled and non-enrolled pre-school children in Busia County, Kenya

Enrolled PSAC had higher prevalence of STH infections, compared to non-enrolled PSAC. However, only difference in prevalence among the two groups of children for *T. trichiura* infection was significant (proportion test: Z = 3.024; *p* = 0.003) (Table 1). Figure 2 provides the STH prevalence among the two groups of PSAC in each school**,** and the surrounding community. The figure shows that the prevalence was greater among enrolled children in three out of the five surveyed schools/communities.

Prevalence of *S. mansoni* infection was 11.8% (95%CI: 11.0–12.9) with mean intensity of 38 epg (95%CI: 36–39). Enrolled PSAC had non-significantly *S. mansoni* infection prevalence than the non-enrolled PSAC (proportion test: Z = 0.350; *p* = 0.727) (Table 1). Figure 2 provides the *S. mansoni* prevalence among the two groups of PSAC in each school**,** and the surrounding community. Similarly, the figure shows that the prevalence was greater among enrolled PSAC in three out of the five surveyed schools/communities.

### Risk factors associated with STH, and *S. mansoni* infections

#### Individual, household, and school WASH characteristics

Table 2 presents the individual WASH characteristics. The average reported number of household occupants was 6.1 people (SD = 2.8 people). Majority (81.0%) of the children were wearing shoes, and geophagy was not common among the PSAC at 18.4%. Nearly half (42.1%) of the children reported to use improved water source for drinking at household level. Reported latrine coverage at household level was high (82.7%). Nevertheless, fewer children reported always having a handwashing facility equipped with water**,** and soap (44.6%) available in their households. Nearly a quarter (19.6%) of the children reported swimming in a dam/river/lake daily.

**Table 2:** Univariable analysis of factors associated with STH and *S. mansoni* infections among pre-school aged children in Busia County, Kenya

Factors	N (%)	STH Combined [OR (95%CI); ***p***-value]	***S. mansoni*** [OR (95%CI); ***p***-value]
Children			
Enrolled	327 (50.1%)	1.38 (0.92–2.09); *p* = 0.123	1.09 (0.68–1.75); *p* = 0.727
Non-enrolled	326 (49.9%)	Reference	Reference
Sex			
Male	330 (50.1%)	Reference	Reference
Female	323 (49.5%)	1.49 (0.98–2.25); *p* = 0.059	0.88 (0.55–1.42); *p* = 0.613
Age group (years)			
3	113 (20.6%)	Reference	Reference
4	145 (26.5%)	1.26 (0.66–2.42); *p* = 0.482	1.38 (0.60–3.12); *p* = 0.456
5	118 (21.5%)	1.08 (0.54–2.16); *p* = 0.834	1.39 (0.59–3.26); *p* = 0.454
> 5	172 (31.4%)	1.35 (0.72–2.52); *p* = 0.349	1.92 (0.89–4.14); *p* = 0.097
Household members			
≤ 5	314 (48.1%)	Reference	Reference
> 5	339 (51.9%)	1.32 (0.88–2.00); *p* = 0.185	1.06 (0.66–1.71); *p* = 0.803
Parent’s education			
No education	279 (42.7%)	2.93 (0.38–22.54); *p* = 0.303	0.74 (0.20–2.67); *p* = 0.646
Primary education	294 (45.0%)	4.33 (0.57–33.11); *p* = 0.158	0.77 (0.21–2.76); *p* = 0.686
Secondary education	61 (9.3%)	5.36 (0.66–43.79); *p* = 0.117	0.28 (0.05–1.50); *p* = 0.136
Post-secondary education	19 (2.9%)	Reference	Reference
Parent’s occupation			
Farming	248 (38.0%)	0.45 (0.17–1.22); *p* = 0.117	0.82 (0.23–2.94); *p* = 0.763
Small business	277 (42.4%)	0.63 (0.24–1.66); *p* = 0.352	1.13 (0.32–3.97); *p* = 0.848
Fishing	72 (11.0%)	1.30 (0.46–3.72); *p* = 0.620	1.47 (0.38–5.69); *p* = 0.580
Employed	25 (3.8%)	Reference	Reference
Roof type			
Grass/Makuti	170 (26.0%)	1.18 (0.75–1.86); *p* = 0.475	0.61 (0.33–1.11); *p* = 0.107
Iron sheets	481 (73.7%)	Reference	Reference
Tiles	2 (0.3%)	Insufficient obs	6.76 (0.42–109.43); *p* = 0.179
Floor type			
Mud/Earthen	410 (62.8%)	0.97 (0.63–1.49); *p* = 0.887	1.01 (0.61–1.65); *p* = 0.989
Cemented	235 (36.0%)	Reference	Reference
Tiles	5 (0.8%)	1.22 (0.13–11.19); *p* = 0.861	Insufficient obs
Wall type			
Mud/Earthen	449 (68.8%)	1.09 (0.69–1.73); *p* = 0.717	1.11 (0.65–1.92); *p* = 0.697
Cemented	183 (28.0%)	Reference	Reference
Blocks/Bricks	20 (3.1%)	0.57 (0.12–2.57); *p* = 0.462	1.44 (0.39–5.34); *p* = 0.587
Wooden	1 (0.2%)	Insufficient obs	Insufficient obs
Latrine in the compound			
Yes	540 (82.7%)	Reference	Reference
No	113 (17.3%)	1.31 (0.79–2.19); *p* = 0.297	0.77 (0.40–1.52); *p* = 0.457
Handwashing facility near latrine			
Yes	241 (44.6%)	Reference	Reference
No	299 (55.4%)	1.27 (0.80–2.02); *p* = 0.317	0.64 (0.38–1.07); *p* = 0.085
Where do you defecate?			
Bush	182 (27.9%)	1.38 (0.89–2.13); *p* = 0.147	0.77 (0.44–1.34); *p* = 0.352
Latrine	455 (69.7%)	Reference	Reference
Ever been dewormed			
Yes	349 (53.5%)	Reference	Reference
No	304 (46.6%)	1.11 (0.74–1.67); *p* = 0.627	0.90 (0.56–1.45); *p* = 0.653
Tablets received when last dewormed			
One	309 (88.5%)	8.63 (1.16–64.16); *p* = 0.035*	0.62 (0.26–1.51); *p* = 0.293
More than one	40 (11.5%)	Reference	Reference
Abdominal pain in the last 2 weeks			
Yes	339 (51.9%)	1.16 (0.77–1.75); *p* = 0.482	0.89 (0.55–1.43); *p* = 0.632
No	314 (48.1%)	Reference	Reference
Diarrhea in the last 2 weeks			
Yes	291 (44.6%)	1.22 (0.81–1.84); *p* = 0.342	0.92 (0.57–1.49); *p* = 0.748
No	362 (55.4%)	Reference	Reference
Improved water source for drinking			
Yes	275 (42.1%)	Reference	Reference
No	378 (38.4%)	2.01 (1.29–3.13); *p* = 0.002*	0.76 (0.47–1.23); *p* = 0.262
Wash fruits before eating			
Yes	402 (61.6%)	Reference	Reference
No	251 (38.4%)	1.45 (0.96–2.19); *p* = 0.075	0.74 (0.45–1.23); *p* = 0.253
Wear shoes/slippers when out of the house			
Never	27 (4.1%)	3.42 (1.14–10.29); *p* = 0.028*	1.23 (0.36–4.18); *p* = 0.738
Sometimes	529 (81.0%)	2.14 (1.04–4.40); *p* = 0.039*	0.92 (0.48–1.79); *p* = 0.813
Always	97 (14.9%)	Reference	Reference
Eat soil			
Yes	120 (18.4%)	1.20 (0.72–1.99); *p* = 0.484	0.55 (0.27–1.15); *p* = 0.111
No	533 (81.6%)	Reference	Reference
Swim in dam/river/lake			
Doesn’t	408 (62.5%)	Reference	Reference
Daily	128 (19.6%)	0.85 (0.48–1.49); *p* = 0.566	2.76 (1.57–4.85); *p* < 0.001*
2–3 times a week	73 (11.2%)	1.96 (1.10–3.49); *p* = 0.023*	2.70 (1.36–5.34); *p* = 0.004*
Weekly	42 (6.4%)	0.86 (0.35–2.13); *p* = 0.750	1.54 (0.57–4.17); *p* = 0.400
More than weekly	2 (0.3%)	Insufficient obs	Insufficient obs

*Indicates a significant *p*-value

### Univariable analysis of factors associated with STH, and *S. mansoni* infections

Univariable analysis of WASH-related factors both at school and household levels revealed significant associations between the infections**,** and some of the covariates of interest as shown in Table 2. For any of the STH infections, the individual factors like never wearing shoes (OR = 3.42 (95%CI: 1.14–10.29), *p* = 0.028), swimming in the river/lake for 2–3 times a week (OR = 1.96 (95%CI: 1.10–3.49), *p* = 0.023), and not using improved water source for drinking (OR = 2.01 (95%CI: 1.29–3.13), *p* = 0.002) increased the odds of STH infections. Notably, taking one deworming tablet (OR = 8.63 (95%CI: 1.16–64.16), *p* = 0.035) showed increased odds of STH infections as opposed to children who took more than one tablet. For *S. mansoni* infections, swimming in dam/river/lake frequently (OR = 2.76 (95%CI: 1.57–4.85), *p* < 0.001) was the only significant risk factor.

### Multivariable analysis of factors associated with STH, and *S. mansoni* infections

Table 3 gives the factors selected for multivariable analysis for STH combined infections. Among the five covariates selected, only two showed statistically significant association with STH infections. PSAC who reported not using improved water source for drinking (aOR = 2.46 (95%CI: 1.26–4.82), *p* = 0.009), and those reported taking only one deworming tablet (aOR = 8.68 (95%CI: 1.13–66.89), *p* = 0.038) showed increased odds of STHs infections.

**Table 3:** Multivariable analysis of factors associated with STH infections among pre-school aged children in Busia County, Kenya

Factors	STH Combined [aOR (95%CI); ***p***-value]
Household members	
≤ 5	Reference
> 5	1.32 (0.73–2.42); *p* = 0.361
Parent’s occupation	
Farming	0.34 (0.09–1.24); *p* = 0.103
Small business	0.50 (0.14–1.75); *p* = 0.279
Fishing	1.09 (0.28–4.35); *p* = 0.899
Employed	Reference
Tablets received when last dewormed	
One	8.68 (1.13–66.89); *p* = 0.038*
More than one	Reference
Improved water source for drinking	
Yes	Reference
No	2.46 (1.26–4.82); *p* = 0.009*
Eat soil	
Yes	1.62 (0.77–3.44); *p* = 0.205
No	Reference

*Indicates a significant *p*-value

Similarly, Table 4 shows factors selected for multivariable analysis for *S. mansoni* infections. Five covariates were selected for multivariable analysis. Only frequent swimming in dam/river/lake showed significant association with *S. mansoni* infections (aOR = 3.99 (95%CI: 1.98–8.06), *p* < 0.001).

**Table 4:** Multivariable analysis of factors associated with *S. mansoni* infections among pre-school aged children in Busia County, Kenya

Factors	***S. mansoni*** [aOR(95%CI); ***p***-value]
Age group (years)	
3	Reference
4	0.74 (0.29–1.88); *p* = 0.521
5	0.76 (0.30–1.95); *p* = 0.568
> 5	1.22 (0.53–2.80); *p* = 0.646
Handwashing facility near latrine	
Yes	Reference
No	0.58 (0.32–1.06); *p* = 0.075
Where do you defecate?	
Bush	1.19 (0.55–2.58); *p* = 0.668
Latrine	Reference
Wear shoes/slippers `when out of the house	
Never	0.95 (0.21–4.23); *p* = 0.943
Sometimes	0.63 (0.30–1.31); *p* = 0.215
Always	Reference
Swim in dam/river/lake	
Doesn’t	Reference
Daily	3.99 (1.98–8.06); *p* < 0.001*
2–3 times a week	2.85 (1.28–6.39); *p* = 0.011*
Weekly	1.99 (0.62–6.40); *p* = 0.247
More than weekly	Insufficient observations

*Indicates a significant *p*-value

## Discussion

This study shows that there was moderate prevalence, and intensity of STH infection among the two cohorts of PSAC with a prevalence of 17.0%, and intensity of 870 (epg). The results corroborate those from studies conducted in Ethiopia which showed that PSAC were also infected with at least one species of STH [36]. This could be due to most PSAC have been left out or neglected in terms of treatment, and control by most school-based deworming programmes (SBDP) over the years. For reasons that they carry insignificant burden of worms. Other studies have found that PSAC have STH infection levels that may require them to be considered for regular treatment [37, 38]. Nevertheless, in the recent years most deworming programmes have started including PSAC for treatment and control of STH. However, there are notable logistical challenges, which the SBDP have yet to overcome, to ensure that all PSAC are included, and participate in SBDP for improved control [39]. Some of the challenges are overcrowding in schools during the deworming day and utilizing primary school teachers or community health extension workers (CHEWs) for deworming. Consequently, there is need to consider using pre-school teachers to deworm PSAC who are familiar with them, to increase coverage, and if possible give them (PSAC) deworming drugs separately from SAC.

It is worth to note that the most prevalent species of STH infection in our current study was *T. trichiura*, followed by *A. lumbricoides* and lastly hookworm*.* This result is contrary to findings from several other studies, which found out that *A. lumbricoides*, and hookworm were the most prevalent species of STH [36, 40]. This could be due to *T. trichiura* is not easily cleared by a single dose of albendazole even after several years of MDA [41]. Accordingly, the need to consider alternative approaches for deworming the PSAC to interrupt transmission such as use of drug combination (albendazole and ivermectin). Nonetheless, the differences in the various study design could have been a contributing factor to variation in outcomes.

Interestingly, the results of our study show that enrolled PSAC where slightly highly infected with STH OR 1.38 (95%CI: 0.92–2.09); *p* = 0.123, and *S. mansoni* OR 1.09 (95%CI: 0.68–1.75); *p* = 0.727 compared to non-enrolled PSAC although it was not statistically significant. This could be due to poor hygiene practices by PSAC while in school. During our visits for data collection to the schools, we observed filled up pit latrines, and unsafe structures which forced kids to defecate around the school compound. This is a possible way of infecting the soil, school environment, and hence the pit latrines could be highly contaminated with STH. In addition, PSAC children could be having contact with fresh water bodies infested with *S. mansoni* during play time especially those above age five.

Assessment of the individual household, school WASH practices and behaviors on STH, and *S. mansoni* infections suggested mixed impacts. Individual-level factors like not wearing shoes was significantly associated with STH infection. In a study conducted in Peru, playing with soil, not wearing sandals, and picking food from the ground were common habits found among PSAC infected with STH [42]. Also, not using improved water source for drinking was significantly associated with STH infection. Other studies have found out that there is an association between STH infection, *S. mansoni* infection, and WASH factors [25, 43, 44]. This necessitates further studies to compare transmission of STH, and *S. mansoni* in school, and home environment.

In the present study, results show that prevalence of STH and *S. mansoni*, increased fairly by age. This agrees with another study which found out that STH infections begun after 3 months of age, and prevalence increased with age [45]. This trend was also observed in other studies conducted elsewhere [46]. This situation might be showing age related change in exposure to STH, and *S. mansoni* infection through playing with soil and shallow waters. Although, most PSAC might not be able to play on their own in outdoor activities, most of them accompany their parents /guardians, and elder siblings while carrying out domestic chores in water bodies. This development factor might explain why infection increases with age.

One limitation of this study was that we used Kato-Katz technique which can miss some egg count in an area with low prevalence especially after several rounds of MDA. We recommend the need for more sensitive diagnostic techniques for programmatic monitoring for STH, and *S.mansoni* [47]**.**

## Conclusion

In conclusion, the present study showed that STH and *S. mansoni* infections are a significant public health problem among PSAC in the study area. This necessitates continued annual deworming, and if possible bi-annual MDA, and combination of albendazole with ivermectin for treatment of *T. trichiura* to control morbidities associated with STH. Some of the associated risk factors in the current study were swimming in the lake/river, not wearing shoes, and using unsafe water source. Hence the need for improved WASH facilities at the school and household levels to reduce environmental contamination with human waste**,** and provision of clean drinking water to control the parasitic diseases among the PSAC. In addition, health education for awareness creation, and behavior change communication on the effects of both parasitic infections needs to be done in schools, and in the villages in future.

## Supplementary information

**Additional file 1.** Questionnaire.

## Data Availability

The datasets analyzed during the current study are available from the corresponding author on reasonable request.

## Abbreviations

KEMRI: Kenya Medical Research Institute
MDA: Mass Drug Administration
NTDs: Neglected Tropical Diseases
NSBDP: National School-Based Deworming Programme
DALYs: Disability-Adjusted Life Years
PSAC: Pre-School Age Children
SAC: School Age Children
SBDP: School-Based Deworming Programme
STH: Soil-Transmitted Helminthes
WHO: World Health Organization
WHA: World Health Assembly
MOH: Ministry of Health
EPG: egg per gram
OR: odds ratio
ECD: Early Childhood Development
WASH: Water Sanitation and Hygiene
ODK: Open Data Kit
SD: Standard Deviation
CI: Confidence Intervals

## References

[1] Molyneux DH, Hotez PJ, Fenwick A (2005). “Rapid-impact interventions”: how a policy of integrated control for Africa’s neglected tropical diseases could benefit the poor. PLoS Med.

[2] Neglected Tropical Diseases: Epidemiology and Global Burden. Trop Med Infect Dis. 2017;2:36. doi:10.3390/tropicalmed2030036. 10.3390/tropicalmed2030036 PMC6082091 30270893

[3] Hotez PJ, Molyneux DH, Fenwick A, Kumaresan J, Sachs SE, Sachs JD (2007). Control of neglected tropical diseases. N Engl J Med.

[4] Hotez P, Bundy D, Beegle K, Brooker S, Drake L (2006). Chapter 24. Helminth Infections: Soil-Transmitted Helminth Infections and Schistosomiasis. Disease Control Priorities in Developing Countries.

[5] Bundy DAP, Cooper ES, Thompson DE, Anderson RM, Didier JM (1987). Age-related prevalence and intensity of Trichuris trichiura infection in a St. Lucian community. Trans R Soc Trop Med Hyg.

[6] Brooker S, Kabatereine NB, Smith JL, Mupfasoni D, Mwanje MT, Ndayishimiye O (2009). An updated atlas of human helminth infections: the example of East Africa. Int J Health Geogr.

[7] Pullan RL, Gething PW, Smith JL, Mwandawiro CS, Sturrock HJW, Gitonga CW (2011). Spatial Modelling of soil-transmitted Helminth infections in Kenya: a disease control planning tool. PLoS Negl Trop Dis.

[8] Crompton DWT, Nesheim MC (2002). Nutritional impact of intestinal helmithiasis during the human lifecycle. Annu Rev Nutr.

[9] World Heath Organization. Neglected Tropical Diseases. 2017. Available, World Health Organization. Neglected Tropical Diseases. Program. 2017.

[10] Carrera E, Nesheim M C, and Crompton DW. Lactose maldigestion in Ascaris-infected preschool children | the American journal of clinical nutrition | Oxford academic. Am J Clin Nutr 1984;39:255–64. https://academic.oup.com/ajcn/article-abstract/39/2/255/4691091. Accessed 27 Feb 2020. 10.1093/ajcn/39.2.255 6695827

[11] Oberhelman RA, Guerrero ES, Fernandez ML, Silio M, Mercado D, Comiskey N (1998). Correlations between intestinal parasitosis, physical growth, and psychomotor development among infants and children from rural Nicaragua. Am J Trop Med Hyg.

[12] Awasthi S, Pande VK (2001). Six-monthly de-worming in infants to study effects on growth. Indian J Pediatr.

[13] De Silva NR, Brooker S, Hotez PJ, Montresor A, Engels D, Savioli L (2003). Soil-transmitted helminth infections: updating the global picture. Trends Parasitol.

[14] World Health Organization. Deworming school-age children Helminth control in school-age children Second edition A guide for managers of control programmes. 2011. http://www.who.int/neglected_diseases/en. Accessed 24 Jan 2020.

[15] Bradbury RS, Graves PM. Current WHO protocols for mass drug administration in helminth control. Microbiol Aust. 2016;37:10–2.

[16] World Health Organization. 17-Seventeenth Programme Report of the UNICEF/UNDP/World Bank/WHO Special Programme for Research & Training in Tropical Diseases. 2005.

[17] World Health Organization. WHO Expert Committee on the Control of Schistosomiasis (2001 : Geneva, Switzerland) & World Health Organization. Prevention and control of schistosomiasis and soil-transmitted helminthiasis : report of a WHO expert committee. 2002. https://apps.who.int/iris/handle/10665/42588. Accessed 11 Sept 2019.

[18] World Health Organization (2012). A roadmap for implementation accelerating work to overcome the global impact of neglected tropical diseases a roadmap for implementation.

[19] World Heath Organization. Investing to Overcome the Global Impact of Neglected Tropical Diseases ... - World Health Organization - Google Books. 2015. https://books.google.co.ke/books?hl=en&lr=&id=mV00DgAAQBAJ&oi=fnd&pg=PR9&dq=WHO.+Investing+to+overcome+the+global+impact+of+neglected+tropical+diseases:+third+WHO+report+on+neglected+diseases+2015.World+Health+Organization+2015.&ots=diyEYXQ_g_&sig=1-zJ4D. Accessed 23 Jan 2020.

[20] Handzel T, Karanja DMS, Addiss DG, Hightower AW, Rosen DH, Colley DG, et al. Geographic distribution of schistosomiasis and soil-transmitted helminths in Western Kenya: implications for anthelminthic mass treatment. Am J Trop Med Hyg. 2003;69:318–23. https://www.ajtmh.org/content/journals/10.4269/ajtmh.2003.69.318. Accessed 11 Sep 2019. 14628951

[21] Brooker S, Kabatereine NB, Gyapong JO, Stothard JR, Utzinger J (2009). Rapid mapping of schistosomiasis and other neglected tropical diseases in the context of integrated control programmes in Africa. Parasitology..

[22] Mwandawiro C, Okoyo C, Kihara J, Simiyu E, Kepha S, Campbell SJ, et al. Results of a national school-based deworming programme on soil-transmitted helminths infections and schistosomiasis in Kenya: 2012-2017. Parasites Vectors. 2019;12. 10.1186/s13071-019-3322-1. 10.1186/s13071-019-3322-1 PMC6367841 30732642

[23] Brooker S, Clements ACA (2009). Spatial heterogeneity of parasite co-infection: determinants and geostatistical prediction at regional scales. Int J Parasitol.

[24] Sousa-Figueiredo JC, Basáñez MG, Mgeni AF, Khamis IS, Rollinson D, Stothard JR (2008). A parasitological survey, in rural Zanzibar, of pre-school children and their mothers for urinary schistosomiasis, soil-transmitted helminthiases and malaria, with observations on the prevalence of anaemia. Ann Trop Med Parasitol.

[25] Roy E, Hasan K, Haque R, … AH-SAJ of, 2011 undefined. Patterns and risk factors for helminthiasis in rural children aged under 2 in Bangladesh. ajol.info. https://www.ajol.info/index.php/sajchh/article/view/73423. Accessed 16 Sept 2019.

[26] Wang X, Zhang L, Luo R, Wang G, Chen Y, Medina A (2012). Soil-transmitted Helminth infections and correlated risk factors in preschool and school-aged children in rural Southwest China. PLoS One.

[27] Mehta RS, Rodriguez A, Chico M, Guadalupe I, Broncano N, Sandoval C, et al. Maternal geohelminth infections are associated with an increased susceptibility to geohelminth infection in children: a case-control study. PLoS Negl Trop Dis. 2012;6. 10.1371/journal.pntd.0001753. 10.1371/journal.pntd.0001753 PMC3404107 22848773

[28] Steinmann P, Utzinger J, Du ZW, Zhou XN. Multiparasitism. A Neglected Reality on Global, Regional and Local Scale. In: Advances in Parasitology. 2010. p. 21–50. doi:10.1016/S0065-308X(10)73002-5. 10.1016/S0065-308X(10)73002-5 20627138

[29] Utzinger J, Bergquist R, Olveda R, Zhou XN. Important Helminth Infections in Southeast Asia. Diversity, Potential for Control and Prospects for Elimination. In: Advances in Parasitology. Academic Press; 2010. p. 1–30. doi:10.1016/S0065-308X(10)72001-7. 10.1016/S0065-308X(10)72001-7 20624526

[30] Olsen A, Samuelsen H, Onyango-Ouma W (2001). A study of risk factors for intestinal helminth infections using epidemiological and anthropological approaches. J Biosoc Sci.

[31] Norhayati M, Oothuman P, Fatmah MS. Some risk factors of Ascaris and Trichuris infection in Malaysian aborigine (orang Asli) children. Med J Malaysia 1998;53:401–7. https://europepmc.org/abstract/med/10971984. Accessed 12 Sept 2019. 10971984

[32] Nyarango RM, Aloo PA, Kabiru EW, Nyanchongi BO (2008). The risk of pathogenic intestinal parasite infections in Kisii municipality, Kenya. BMC Public Health.

[33] Mwinzi PNM, Montgomery SP, Owaga CO, Mwanje M, Muok EM, Ayisi JG, et al. Integrated community-directed intervention for schistosomiasis and soil transmitted helminths in western Kenya - a pilot study. Parasites Vectors. 2012;5. 10.1186/1756-3305-5-182. 10.1186/1756-3305-5-182 PMC3447651 22937890

[34] Kenya National Bureau of Statistics. Kenya Population and Housing Census Analytical Reports. Kenya National Bureau of Statistics. 2009. https://www.knbs.or.ke/2009-kenya-population-and-housing-census-analytical-reports/. Accessed 25 June 2019.

[35] Miguel E, Kremer M (2004). Worms: identifying impacts on education and health in the presence of treatment externalities. Econometrica..

[36] Shumbej T, Belay T, Mekonnen Z, Tefera T, Zemene E. Soil-Transmitted Helminths and Associated Factors among Pre-School Children in Butajira Town, South-Central Ethiopia: A Community-Based Cross-Sectional Study. PLoS One. 2015;10. 10.1371/journal.pone.0136342. 10.1371/journal.pone.0136342 PMC4548951 26305361

[37] Davis SM, Worrell CM, Wiegand RE, Odero KO, Suchdev PS, Ruth LJ (2014). Soil-transmitted helminths in pre-school-aged and school-aged children in an urban slum: a cross-sectional study of prevalence, distribution, and associated exposures. Am J Trop Med Hyg..

[38] Novianty S, Dimyati Y, Pasaribu S, Pasaribu AP. Risk factors for soil-transmitted Helminthiasis in preschool children living in farmland, north Sumatera, Indonesia. J Trop Med. 2018. 10.1155/2018/6706413. 10.1155/2018/6706413 PMC5904775 29849666

[39] Musuva RM, Matey E, Masaku J, Odhiambo G, Mwende F, Thuita I (2017). Lessons from implementing mass drug administration for soil transmitted helminths among pre-school aged children during school based deworming program at the Kenyan coast. BMC Public Health.

[40] Kirwan P, Asaolu SO, Molloy SF, Abiona TC, Jackson AL, Holland CV (2009). Patterns of soil-transmitted helminth infection and impact of four-monthly albendazole treatments in preschool children from semi-urban communities in Nigeria: a double-blind placebo-controlled randomised trial. BMC Infect Dis.

[41] Hall A, Nahar Q (1994). Albendazole and infections with Ascaris lumbricoides and Trichuris trichiura in children in Bangladesh. Trans R Soc Trop Med Hyg.

[42] Omitola OO, Mogaji HO, Oluwole AS, Adeniran AA, Alabi OM, Ekpo UF. Geohelminth Infections and Nutritional Status of Preschool Aged Children in a Periurban Settlement of Ogun State. Scientifica (Cairo). 2016;2016. 10.1155/2016/7897351. 10.1155/2016/7897351 PMC4789517 27034905

[43] Asaolu SO, Ofoezie IE, Odumuyiwa PA, Sowemimo OA, Ogunniyi TAB (2002). Effect of water supply and sanitation on the prevalence and intensity of Ascaris lumbricoides among pre-school-age children in Ajebandele and Ifewara, Osun state, Nigeria. Trans R Soc Trop Med Hyg.

[44] Babatunde TA, Asaolu SO, Sowemimo OA. Urinary schistosomiasis among pre-school and school aged children in two peri-urban communities in Southwest Nigeria. http://citeseerx.ist.psu.edu/viewdoc/download?doi=10.1.1.680.403&rep=rep1&type=pdf. Accessed 14 Mar 2020.

[45] Menzies SK, Rodriguez A, Chico M, Sandoval C, Broncano N, Guadalupe I, et al. Risk factors for soil-transmitted Helminth infections during the first 3 years of life in the tropics; findings from a birth cohort. PLoS Negl Trop Dis. 2014;8. 10.1371/journal.pntd.0002718. 10.1371/journal.pntd.0002718 PMC3937274 24587469

[46] Sowemimo OA, Asaolu SO. Current status of soil-transmitted helminthiases among pre-school and school-aged children from Ile-Ife, Osun state, Nigeria. J Helminthol. 2011;85:234–8. 10.1017/S0022149X10000489 20810012

[47] Levecke B, Behnke JM, Ajjampur SSR, Albonico M, Ame SM, Charlier J, et al. A comparison of the sensitivity and fecal egg counts of the McMaster egg counting and Kato-Katz thick smear methods for soil-transmitted helminths. PLoS Negl Trop Dis. 2011;5. 10.1371/journal.pntd.0001201. 10.1371/journal.pntd.0001201 PMC3114752 21695104

