# Supplementary material for: Soil-transmitted helminths and schistosomiasis among pre-school age children in a rural setting of Busia County, Western Kenya: a cross-sectional study of prevalence, and associated exposures
Source: BMC Public Health. 2020 Mar 18;20:356. doi: 10.1186/s12889-020-08485-z (PMC7079432; doi:10.1186/s12889-020-08485-z)
Supplement: Supplementary file 1 — Additional file 1. Questionnaire. [file 12889_2020_8485_MOESM1_ESM.docx]

**Questionnaire**

**Identification section**

ID code (respondent) .............................................Village/School Name...............................

House No.......................................... Date ......./......../...........

Name of interviewer......................... Stool taken 1. Yes............ 2. No..............

**Demographic section**

1. Sex of the child

- M……………………
- F……………………

1. Age of the child (in years) ..................
2. Name of parent/guardian………………………….
3. How many people live in your house?...............
4. What is your parent/guardian education level?

- None
- Primary school
- Secondary school
- College/ university

1. What is your guardian/parent occupation?

- Farming
- Small business
- Fishing
- Employed
- Others (specify)………………………

1. House roof type

- Grass/Makuti(thatched)
- Iron sheets
- Tiles
- Others specify

1. House floor type

- Mud/ earthen
- Cemented
- Tiles
- Others specify

1. Do you have a latrine in your compound?

- Yes
- No

1. If yes, do you have hand washing facility near the latrine?

- Yes
- No

1. When need arise for natural call where do you normally go?

- Bush
- Latrine
- Others specify…………………….

1. Have you ever been dewormed?

- Yes
- No

1. If yes, when was the last time you were dewormed?

- More than 1 year ago
- More than 6 months ago
- More than 3 months ago
- Within one month
- I don’t remember
- Never dewormed

1. Where were you dewormed lastly?

- School
- Home
- Hospital
- Others specify

1. How many tablets did you receive?................................
2. Who did the deworming?

□ Teacher

□ Parent/guardian

□ Nurse/Health worker

□ Others specify………………………………………

1. During the last 2 weeks have you experienced any abdominal pains?

- Yes
- No

1. During the last 2weeks did you have an onset of diarrhea?

- Yes
- no

1. What is the main source of drinking water in your house hold?

- Dam
- River
- Rain water
- Tap water
- Others specify

1. Do you usually wash fruits before eating?

- Yes
- No

1. How often do you wear shoes or slippers when out of the house?

- Never
- Sometimes
- Always

1. When do you remove your shoes?

- While playing
- Rainy day
- While on farm
- While in the house
- Others specify

1. Do you eat clay/soil?

- Yes
- No

1. If yes, when is the last time you ate clay/soil?

- More than 1 year ago
- More than 6 months ago
- More than 3 months ago
- Within one month
- Within 2 weeks
- I don’t remember

1. How often do you bath swim in dam/river or along the lake?

- Daily
- 2-3 times a week
- Weekly
- Others specify

**Supervisors signature……………………… Date………………….**

**Investigator's signature: ……………………… Date……………..........**
